# Comparative study of precise measurements of natural radionuclides and radiation dose using *in-situ* and laboratory γ-ray spectroscopy techniques

**DOI:** 10.1038/s41598-018-32220-9

**Published:** 2018-09-20

**Authors:** N. M. Hassan, Y. J. Kim, J. Jang, B. U. Chang, J. S. Chae

**Affiliations:** 1 Department of Natural Radiation Safety, Korea Institute of Nuclear Safety, 62, Gwahak-ro, Yuseong-gu, Daejeon, 34142 Republic of Korea; 2 Department of Physics, Faculty of Science, Zagazig University, PO Box 44519, Zagazig, Egypt

## Abstract

In this study, *in-situ* and laboratory γ-ray spectroscopy techniques were compared to evaluate the activity concentration of natural radionuclides in soil. The activity concentrations of ^238^U (^226^Ra), ^232^Th, and ^40^K in the soil in 11 sites were simultaneously measured with *in-situ* portable HPGe and the NaI(Tl) detectors. In parallel, 55 soil samples collected from these sites were analyzed with a laboratory γ-ray spectroscopy technique (HPGe). A strong correlation was observed between the *in-situ* and laboratory HPGe techniques with a linear correlation coefficient (R^2^) of 0.99 for ^226^Ra and ^232^Th and 0.975 for ^40^K, respectively. The *in-situ* HPGe technique shows a strong correlation with the NaI(Tl) detector. γ-Rays cps of ^226^Ra, ^232^Th, and ^40^K of the NaI (Tl) detector were then converted to specific activities (Bq kg^−1^ unit) in soil using the empirical formulas obtained in this study. The absorbed dose rate in air at 1 m height above ground due to these radionuclides was calculated using the Beck’s formula and the results were compared with measured values obtained with an high pressure ionization chamber. The results of the calculated and measured dose rate show a strong correlation of R^2^ = 0.96. The reliability and precision of analytical spectroscopy techniques of radioactivity and radiation dose were confirmed in this work.

## Introduction

Human beings are continuously exposed to ionizing radiation emitted from natural radionuclides maintained in the earth’s crust or soil. The soil is considered as the main source of natural radionuclides of ^238^U, ^232^Th, ^40^K, and radon gas^1–3^. About 80% of delivering radiation dose is due to the natural radionuclides^2^. The received radiation dose to human beings mainly depends on the concentrations of these radionuclides. This radiation dose varies over the whole world, depending on the variation of radionuclides concentration in the geological constructions^4^. Recently, several countries have been conducting surveys to evaluate the radiation dose in their location in order to provide nationwide data of radiation background to act as a reference data source, and in case of contamination, it can support decision making^5^. Korea is also conducting a survey to evaluate the radiation background of natural radionuclides for in a radon potential mapping program. The most common methods to evaluate both the natural radionuclides and the dose are *in-situ* NaI(Tl) and HPGe (High Purity Germanium) γ-ray spectrometry, and HPIC (High-Pressure Ionization Chamber) measurement^6,7^.

The *in-situ* γ-ray spectroscopy technique is a quick and low-cost method to evaluate the activity concentration of natural radionuclides in soil. NaI(Tl) and HPGe detectors are used for *in-situ* γ-ray spectrometry. The NaI(Tl) detector has advantages over portable systems including relatively high efficiency, easy operation, and maintenance, etc. Based on these advantages, portable γ-ray spectrometry using the NaI(Tl) detector has been well-developed and has been used for uranium exploration, geological mapping, and environmental studies since the 1960’s^8^. A NaI(Tl) detector equipped in a vehicle, that is car-borne γ-ray spectrometry, provides much greater coverage for a given time and cost and is used for regional and detailed mapping surveys for estimating the surface concentrations of the radionuclides^9^. Meanwhile, in the past 30 years, the *in-situ* portable HPGe detector was used to measure the radionuclides in soil on the basis of its high resolution compared to NaI(Tl) as well as its precise detection^10^. To determine the surface concentrations of the radionuclides, the sample is assumed to be a flat, unimpeded surface, in which the horizontal distribution of the radionuclides is practically uniform^11^.

The measured concentrations of natural radionuclides using *in-situ* NaI(Tl) γ-ray spectrometry are determined through measurements of the naturally occurring terrestrial gamma radiation of ^40^K (1460.8 keV) and the decay series of ^232^Th (at 2614.5 keV of ^208^Tl) and ^238^U (at 1764.5 keV of ^214^Bi)^6^. According to the IAEA documents^9,12^, the *in-situ* NaI(Tl) γ-ray spectrometer is calibrated by means of calibration pads. A calibration pad is a slab of concrete containing known concentrations of the radionuclides. The background of the detectors also should be estimated. Another calibration method is comparing the potassium, uranium, and thorium window count rates over a calibration site with the ground concentrations of potassium, uranium, and thorium measured with a calibrated portable γ-ray spectrometer^13^. To carry out the calibration using this method, it is necessary to measure these nuclides in advance, check the homogeneity of the concentration distributions in the calibration site, and determine the background count rate of the car-borne γ-ray spectrometry system.

Recently, Korea Institute of Nuclear Safety (KINS) has been carrying out a nationwide terrestrial gamma radiation survey using car-borne γ-ray spectrometry in order to map the terrestrial radiation as the radon source term and the geogenic radon potential risk in Korea. In this study, a convenient calibration method for car-borne γ- ray spectrometry has been discussed using a comparison of *in-situ* and laboratory γ-ray spectroscopy techniques without previous characterization of the calibration sites and determination of the background of the system, as a part of the nationwide terrestrial radiation survey. To accomplish this, the measurements of natural radionuclides (^40^K, ^238^U, ^232^Th) with car-borne measurement and *in-situ* and laboratory γ-ray spectroscopy techniques have been compared in 11 sites with different natural radiation levels. At the same time, the γ-ray absorbed dose rate was measured with a high-pressure ionization chamber (HPIC) detector and the results were compared with values calculated using the radionuclide concentrations. The car-shielding effect of the HPIC detector was also discussed.

## Experimental Technique

### Measurement setup and sample preparation

**Figure 1:**
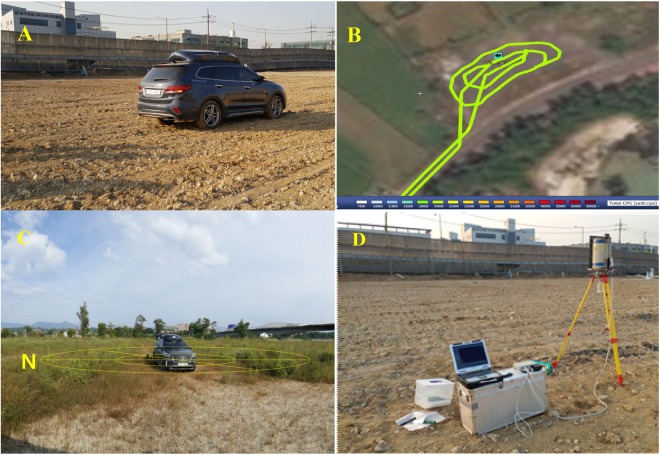
Pre-setting of gamma spectroscopic techniques of *in-situ* NaI(Tl) detector in the survey vehicle and *in-situ* HPGe detector. Colored line (**B**) represents the levels of total γ-ray count rate of NaI(Tl) detector automatically generated by the operating software (TRMS, Terrestrial γ-Radiation Monitoring System, made by RadSearch Co., Korea) of the car-borne measurement system on the satellite image of cesium (http://cesiumjs.org).

The *in-situ* measurement was carried out using two processes, γ-ray counts with a NaI(Tl) detector fixed in a car-borne survey system and an *in-situ* HPGe portable detector. First, the car-borne survey was performed to measure the whole selected site to check the homogeneity of natural radionuclide distributions in the soil and the flatness of the open area (no buildings) within 400 m^2^ from the measurement site, as seen in Fig. 1A. To check the homogeneity, the vehicle moved with a very low speed of less than 2 km/h. At the same time, γ-ray counts were measured with the NaI(Tl) detector. The site within 10% of total count per second (cps) variation of each 10 second NaI(Tl) spectrum was selected for calibration, as shown in Fig. 1B, which is a satellite image (obtained from an open-source JavaScript library, cesium^®^ (http://cesiumjs.org)) combined with car-borne survey results expressed with colors indicating total count rate of gamma ray using the operating software (RadSearch Co., Korea) of the car-borne measurement system.

**Figure 2:**
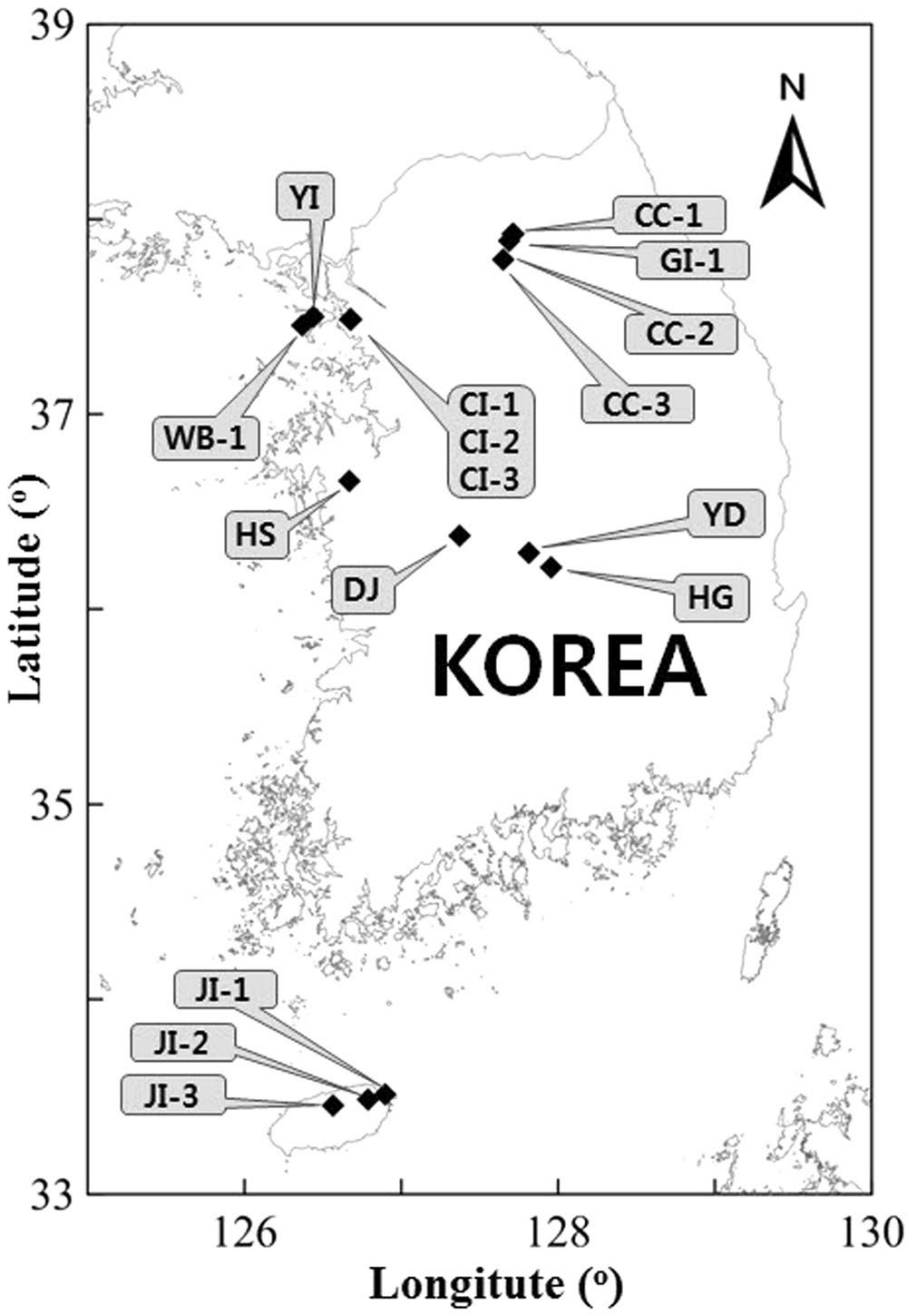
Location of the measured sites in this study.

After checking the homogeneity, the car-borne system was fixed at the center of the radiologically homogeneous area in the site and measured γ-rays counts of ^238^U, ^232^Th, and ^40^K in the soil for 10 minutes. 600 spectra acquired for 10 minutes were cumulated into a single spectrum, which was used for spectrum analysis. After car-borne measurement, *in-situ* HPGe measurement and soil sampling were carried out at five points (the center of car-borne measurement position, 5 m circle diameter (east and west) and 10 m circle diameter (north and south)), as shown in Fig. 1C. At each of those five points, the activity concentrations of ^238^U (^226^Ra), ^232^Th, and ^40^K measured with the *in-situ* HPGe portable detector were averaged as the specific activity concentrations of natural radionuclides of ^238^U (^226^Ra), ^232^Th, and ^40^K in this site, as given in Fig. 1D. Five soil samples (surface thickness of 10 cm) taken at the same points were sent to KINS laboratory for laboratory γ-ray spectroscopy analysis. Based on data from on-going nationwide terrestrial gamma radiation survey, total 11 sites were selected to cover all the varieties of natural radionuclide content in soil across Korea, as shown in Table 1 and Fig. 2.

**Table 1:** Characteristic parameters of selected sites.

Site name	Sits code	Locations	Geological origin	Total cps of NaI(Tl)	Latitude	Longitude	Measurement
Site 1	JI-1	Jeju island	Quarternary Basalt/trachyte	1,500	33°27′13.8″	126°33′59.3″	NaI(Tl), *in-situ* HPGe, soil sampling, HPIC
Site 2	JI-2	Jeju island	Quarternary Basalt	917	33°29′6.5″	126°47′27.4″	NaI(Tl), *in-situ* HPGe, soil sampling, HPIC
Site 3	JI-3	Jeju island	Quarternary Basalt	789	33°30′51.0″	126°53′56.1″	NaI(Tl), *in-situ* HPGe, soil sampling, HPIC
Site 4	CI-1	Incheon	Quarternary Alluvium (NORM contaminated area)	2,950	37°29′11.4″	126°40′33.5″	NaI(Tl), *in-situ* HPGe, soil sampling,
Site 5	CI-2	Incheon	Quarternary Alluvium (NORM contaminated area)	5,077	37°29′15.0″	126°40′37.1″	NaI(Tl), *in-situ* HPGe, soil sampling,
Site 6	CI-3	Incheon	Quarternary Alluvium (NORM contaminated area)	3,738	37°29′14.2″	126°40′37.1″	NaI(Tl), *in-situ* HPGe, soil sampling,
Site 7	GI-1	Gosmdochi island	Quarternary Alluvium	2,654	37°55′22.8″	127°42′59.0″	NaI(Tl), *in-situ* HPGe, soil sampling,
Site 8	CC-1	Chuncheon	Jurassic Granite	6,184	37°55′20.7″	127°42′44.6″	NaI(Tl), *in-situ* HPGe, soil sampling,
Site 9	CC-2	Chuncheon	Jurassic Granite	3,144	37°53′16.9″	127°41′10.9″	NaI(Tl), *in-situ* HPGe, soil sampling,
Site 10	CC-3	Chuncheon	Jurassic Granite	2,706	37°47′29.1″	127°39′5.1″	NaI(Tl), *in-situ* HPGe, soil sampling,
Site 11	WB-1	Wangsan beach	Jurassic Granite	4,765	37°27′17.9″	126°22′149″	NaI(Tl), *in-situ* HPGe, soil sampling,
Site 12	DJ	Daejeon	Jurassic Granite	1,990	36°22′36.9″	127°22 21.2″	HPIC
Site 13	YD	Yeongdong	Triassic Porphyry Granite	3,175	36°17′21.2″	127°48 55.3″	HPIC
Site 14	HS	Hongseoung	Jurassic Granite	3,366	36°39′27.6″	126°40 9.9″	HPIC
Site 15	YI	Yeonjong island	Reclaimed land	5,376	37°29′53.6″	126°26 22.5″	HPIC
Site 16	HG	Hwang-gan	Precambrian Gnesis	6,764	36°12′46.1″	127°57 13.0″	HPIC

A total of 55 soil samples were collected from 11 sites over Korea, seen in Table 1 and Fig. 2. The selected samples were crushed into a fine powder. These samples were crushed without removing any of their content, except for plant root debris, etc. to preserve the radioactivity level. They were then sieved through a 1 mm mesh size to be more homogenous. The sample was then dried in an oven of controlled temperature at 110 °C for 24 hours to ensure that moisture is completely removed. After moisture removal, the samples were cooled to room temperature in a desiccators. The prepared samples were packed into airtight plastic cylindrical containers (6 cm diameter and 4 cm height) made from polyethylene. The containers were carefully sealed with an adhesive to prevent any possibility of radon (^222^Rn) or thoron (^220^Rn) escaping and stored for one month to achieve radioactive secular equilibrium between radium and radon^14^. At the same time, an empty container, with the same geometry as used for the samples, was also sealed and left for the same time in order to be used for background measurement.

### Car-borne gamma-ray spectrometry

#### Car-borne instrumentations

The car-borne instrumentations for measuring the natural radionuclides concentrations of ^226^Ra (^238^U), ^232^Th, and ^40^K, as well as the ambient dose rate, consisted of a large volume NaI(Tl) detectors (4″ × 4″ × 16″, SAINT-GOBAIN), a multi-channel analyzer (MCA, ORTEC Co.), a High Pressure Ionization Chamber (25.4 cm ϕ, HPIC RS Detection, General Electric Co.) environmental radiation monitor, a global position system (GPS) terminal (GNSS, AscenKorea Co.), a long-term evolution (LTE) wireless communication system, and an operating software (RadSearch Co.). For the normal survey, the vehicle moved at low speed, less than 60 km/h, and at the same time, γ-ray spectrum, ambient dose rate, and GPS signal were continuously recorded with the Na(Tl) detector, the HPIC detector, and the GPS terminal, respectively. Each measurement time was adjusted to be 10 seconds. All data were collected every 10 seconds and sent in real time to the KINS main sever. The surveyed routes were fed back to the operating computer in the vehicle at the almost same time to avoid the duplicate survey points. In this study, the vehicle’s measurement time was adjusted to be 10 minutes.

#### Data processing for NaI(Tl) spectrum after the car-born measurement

After the car-borne measurements, the 600 spectra measured in 10 minutes at each measurement site were cumulated into a single spectrum. The cps of ^40^K (1460.8 keV), ^238^U (^226^Ra via ^214^Bi (1764.5 keV)), and ^232^Th (via ^208^Tl (2614 keV) were extracted from the cumulated gamma spectrum using the method described by the IAEA report TECDOC-1363^9^. These cps values of ^40^K, ^238^U, and ^232^Th were compared to the activity concentration of the same radionuclides measured with the *in-situ* portable HPGe spectrometer at the same measurement site.

#### Correction of radiation dose values measured by the HPIC detector

The shielding attenuation effect of γ-rays within the vehicle’s materials was measured in order to correct radiation dose values that were measured by the HPIC detector. For this purpose, several open and flat areas were selected using the results of the nationwide terrestrial radiation survey as mentioned before. The radiation dose rate was measured while the HPIC detector was equipped in the vehicle for 10 minutes. It was then measured again at the same point for the same time while the same HPIC detector was outside the vehicle. In both cases, the HPIC detector was set up at 1 m height from the ground. The measured dose rates with the HPIC detector inside and outside the vehicle were compared. An equation was obtained from the correlation between the dose rate measured inside and outside the vehicle. From that equation, the shielding factor was estimated.

The HPIC detector used in this study was calibrated by the manufacturer. For more precise measurement, this HPIC detector was compared with another HPIC detector calibrated on 12^th^ October 2017, by Korea Research Institute of Standards and Science (KRISS), at the same measurement points. The obtained results for measuring dose values with both of these HPIC detectors show a deviation of less than 1%.

### *In-situ* HPGe spectroscopy technique

Natural radionuclides concentrations of ^226^Ra, ^232^Th, and ^40^K were measured using an *in-situ* portable p-type HPGe detector manufactured by a Canberra model GC3018 with an efficiency of 30% and an energy resolution of 1.9 keV at 1.33 MeV of the γ-ray line of ^60^Co. The detector was housed in an aluminum end cap and attached to a small portable crystal of 7 L of liquid nitrogen. The electronics of a high voltage supply, amplifier, analog-digital converter, and multichannel analyzer were highly integrated into a small unit attached to the system^5,11^. The measurement spectra were displayed and stored on a notebook computer. The *in-situ* HPGe detector calibration and efficiency were estimated using semi-empirical techniques proposed in the literature by Helfer and Miller, and Baeza *et al*.^11,15^. The measurement time was proposed to be 2400 seconds in order to acquire a clear spectrum. The HPGe portable detector was fixed at 1 m height facing toward the ground. All measurements were carried out on dry days to minimize the shielding effect of soil moisture.

The radionuclide activity concentrations of ^226^Ra and ^232^Th were measured from γ-rays emitted from their daughters of ^214^Pb and ^214^Bi for ^226^Ra and ^228^Ac and ^208^Tl for ^232^Th while for ^40^K its own γ-ray was used. Radium (^226^Ra) specific activity concentration was measured from the γ-rays with the energy of 351.9 keV associated with the decay ^214^Pb, and 609.3 keV, 1120 keV and 1764.5 keV γ-rays associated with the decay of ^214^Bi. Thorium (^232^Th) specific activity concentration was estimated from the γ-rays of energies of 911.1 keV and 968.8 keV associated with the decay of ^228^Ac, and 583.1 keV and 2614.5 keV associated with the decay of ^208^Tl. Potassium (^40^K) specific activity concentration was estimated from the γ-ray with the energy of 1460.9 keV associated with the decay of ^40^K. The estimated activity concentrations were compared with the values obtained using the laboratory γ-ray spectroscopy technique.

### Laboratory HPGe spectroscopy technique

Natural radionuclide concentrations of ^226^Ra, ^232^Th, and ^40^K were measured using a vertical closed-end coaxial HPGe detector manufactured by ORTEC (model number GEM60-83-XLB-C) with an efficiency of 60% and an energy resolution of 1.95 keV at 1.33 MeV of γ-ray line of ^60^Co. The detector was shielded with a cylindrical lead with a thickness 10 cm that contains an inner concentric cylinder of Cu with a thickness of 8 mm in order to reduce the background effects. It was connected to a personal computer-based data acquisition system that has a Multi-Channel-Analyzer (ORTEC, DSPEC-50, digital spectrometer of DPEC-502-KT). The data analysis was carried out via a gamma spectroscopy program of ORTEC GammaVison software, model A66-B32 and version 6.09. The mass-metric efficiency and spectra energies calibration were calculated using a liquid standard source (commercial name of TCC mixture source) containing several radionuclides of (^241^Am, ^109^Cd, ^57^Co, ^139^Ce, ^203^Hg, ^113^Sn, ^85^Sr, ^134^Cs, ^137^Cs, ^54^Mn, ^88^Y, and ^65^Zn) certified by Eckert and Ziegler. The standard source was chosen to be TCC to overcome the true coincidence summing correction in the efficiency calculation and consequently the specific activity concentrations of different radionuclides. Moreover, the results were validated against the certified reference soil material of IAEA-447.

Since radium (^226^Ra) and its progenies produced about 98.5% of radiological effects of uranium series, the contribution of ^238^U and the precursors of ^226^Ra were ignored^16,17^. Thus, radium (^226^Ra) was considered to be the reference of the ^238^U series instead of ^238^U. The radium (^226^Ra) specific activity concentration was measured from γ-rays of energies of 351.9 keV (35.1%) associated with the decay of ^214^Pb and 609.3 keV (46.6%) and 1120 keV (14.7%) γ-rays associated with the decay of ^214^Bi. The thorium (^232^Th) specific activity concentration was estimated from γ-rays of energies of 911.1 keV (26.6%) and 968.8 keV (16.23) associated with the decay of ^228^Ac and 583.1 keV (30.6%) associated with the decay of ^208^Tl. The potassium (^40^K) specific activity concentration was estimated from the γ-ray’s energy of 1460.9 keV (10.67%) associated with the decay of ^40^K. The specific activity concentration of these natural radionuclides, *A*, (Bq kg^−1^) was calculated from Eq. (1) ^14,18^.

1 $$A=\frac{C}{pwt\varepsilon }$$

where *C* is the net count above the background, *p* is the absolute emission probability of the gamma ray (mentioned in brackets after γ-rays energies), *w* is the net dry sample weight (kg), *t* is the measurement time which was adjusted for all measurements to be 80000 seconds, and *ε* is the absolute efficiency of the detector.

## Results and Discussion

### Evaluation of specific activity concentration of natural radioactive nuclides

The Earth’s crust is the main source of terrestrial radiation dose due to the presence of measurable amounts of natural radioactive nuclides of ^238^U, ^232^Th, their progenies, and ^40^K, which vary corresponding to the variation of geological origin and the received dose to human beings as well^3^. Therefore, precise measurement of these natural radionuclides is very important to protect human beings from the harmful effects of ionizing radiation. Korea has paid attention to the accurate evaluation of the activity concentrations of natural radionuclides and their corresponding radiation dose over its area.

Activity concentrations of natural radionuclides of ^226^Ra(^238^U), ^232^Th, and ^40^K in 11 sites across Korea were measured using *in-situ* gamma spectroscopy techniques (portable HPGe detector), as shown in Tables 1 and 2. The concentrations varied from 13.4 ± 0.7 to 188 ± 21, 14.6 ± 0.1 to 230 ± 4, and 241 ± 4 to 1,328 ± 8 Bq kg^−1^ with corresponding mean values of 61.4 ± 5, 76.2 ± 5.6, and 808 ± 32 Bq kg^−1^, respectively, as shown in Table 2. It was noted that all natural radionuclide concentrations were lower on Jeju Island due to its geological origin of Quarternary Basalt/trachyte while they were higher in Chuncheon due to its geological origin of Jurassic granite, as shown in Table 1. The presented data were consistent with the published data in the literature^19^.

**Table 2:** Activity concentration of natural radionuclides that measured with *in-situ* and laboratory γ-rays spectroscopy techniques.

Site code	Activity concentration of radionuclides (Bq kg^−1^)								
	^226^Ra			^232^Th			^40^K		
	*In-situ*	Laboratory	*In-situ*/Lab.	*In-situ*	Laboratory	*In-situ*/Lab.	*In-situ*	Laboratory	*In-situ*/Lab.
JI-1	22.9 ± 2.0	25.0 ± 1.1	0.92 ± 0.09	37.6 ± 2.7	40.3 ± 1.3	0.93 ± 0.07	630 ± 15	713 ± 14	0.88 ± 0.03
JI-2	21.4 ± 6.1	16.9 ± 0.8	1.26 ± 0.36	22.0 ± 1.2	22.7 ± 1.0	0.97 ± 0.07	276 ± 8	223 ± 12	1.24 ± 0.08
JI-3	13.4 ± 0.7	9.8 ± 0.3	1.37 ± 0.08	14.6 ± 1.0	13.0 ± 0.4	1.12 ± 0.08	241 ± 4	291 ± 12	0.83 ± 0.04
CI-1	53.9 ± 2.0	48.9 ± 2.3	1.10 ± 0.07	76.3 ± 1.7	65.6 ± 1.3	1.16 ± 0.03	806 ± 11	721 ± 15	1.12 ± 0.03
CI-2	188 ± 21	214 ± 13.8	0.88 ± 0.11	101 ± 8	99.5 ± 8.0	1.01 ± 0.11	868 ± 44	844 ± 29	1.03 ± 0.06
CI-3	63.1 ± 1.3	68.6 ± 2.8	0.92 ± 0.04	99.2 ± 1.5	95.2 ± 2.2	1.04 ± 0.03	1060 ± 14	1001 ± 26	1.06 ± 0.03
GI-1	31.9 ± 1.2	27.2 ± 1.4	1.17 ± 0.07	68.2 ± 1.8	68.8 ± 1.6	0.99 ± 0.03	787 ± 19	811 ± 27	0.97 ± 0.04
CC-1	146 ± 5	166 ± 2	0.88 ± 0.03	230 ± 4	236 ± 10	0.97 ± 0.04	1328 ± 9	1390 ± 28	0.96 ± 0.02
CC-2	38.9 ± 4.3	36.2 ± 2.6	1.08 ± 0.14	76.0 ± 3.1	69.9 ± 0.7	1.09 ± 0.05	980 ± 9	974 ± 9	1.01 ± 0.01
CC-3	34.9 ± 3.2	30.9 ± 1.0	1.13 ± 0.11	37.0 ± 2.1	30.4 ± 0.6	1.22 ± 0.07	1098 ± 8	1041 ± 12	1.06 ± 0.01
WB-1	60.5 ± 5.9	73.1 ± 5.6	0.83 ± 0.10	168 ± 14	173 ± 15	0.97 ± 0.12	1159 ± 13	1159 ± 28	1.00 ± 0.03

All uncertainties were combined uncertainties at k = 1 given by the gamma spectroscopy programs of Gene2000 and Gamma Vision software.

**Figure 3:**
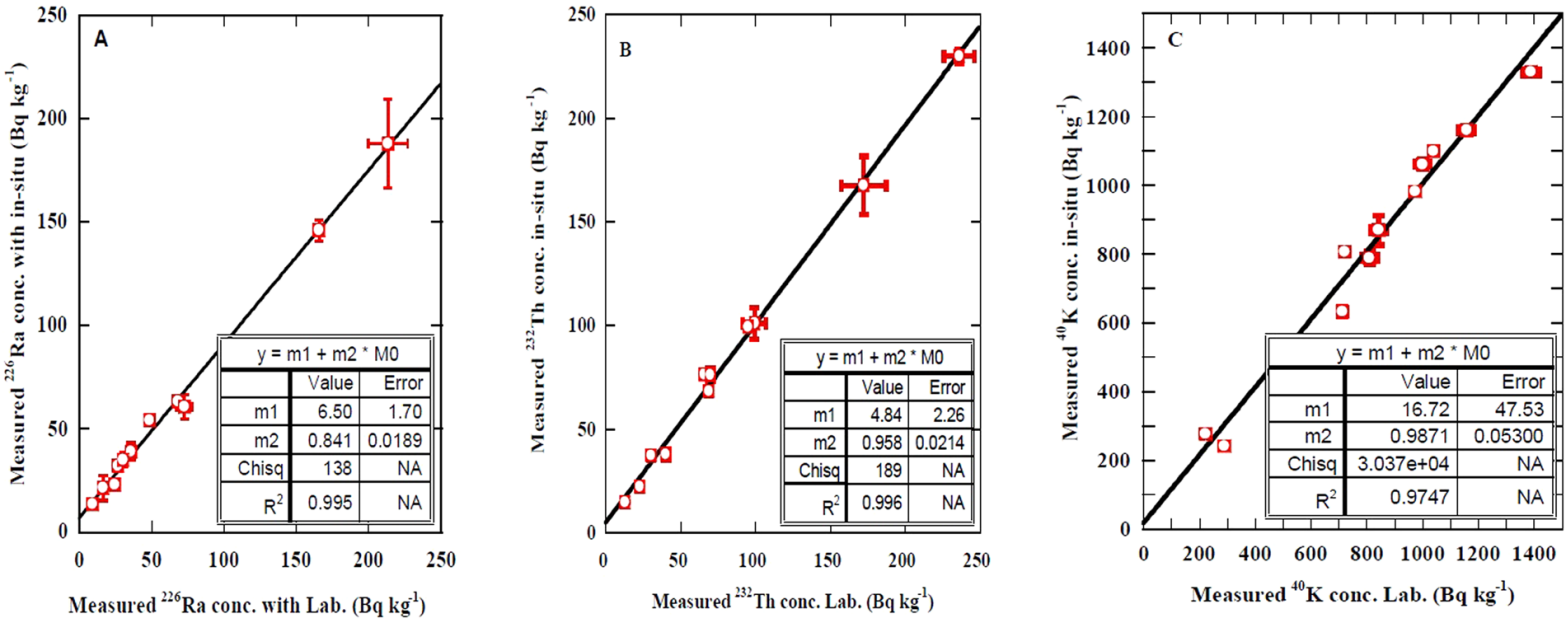
The relation of measured activity concentration of ^226^Ra, ^232^Th and ^40^K using *in-situ* and laboratory gamma spectroscopy techniques (both using HPGe detectors).

To achieve good quality assurance for the measured values, the specific activity concentrations of natural radionuclides in 55 samples from 11 sites over the whole Korean area (5 samples from each site) were measured with a well calibrated conventional laboratory gamma spectrometer (HPGe)^11^. A comparison between the measured values of ^226^Ra (^238^U), ^232^Th, and ^40^K with the two techniques of *in-situ* and laboratory γ-ray spectrometers are presented in Fig. 3 and Table 2, and the ratio is shown as well. The difference between the two techniques was less than 1% for ^232^Th and ^40^K, while it was within 15% for ^226^Ra, due the effect of radon in the case of the *in-situ* technique^4,5,11^. A strong correlation between these techniques can be clearly observed in Fig. 3 with linear regression lines of 99% for ^226^Ra and ^232^Th whereas for ^40^K, the correlation was 97.5% and the corresponding line equations were y = (0.84 ± 0.02) x + (6.5 ± 1.7), y = (0.96 ± 0.02) x + (4.8 ± 2.3), and y = (0.99 ± 0.05) x + (16 ± 47), respectively, as seen in Fig. 3. This study shows much better correlations between *in-situ* and laboratory gamma spectrometers (HPGe detector) than the published data of Baeza *et al*.^11^ who reported the correlation of *in-situ* and laboratory gamma spectrometers was 0.94 for ^40^K and ^232^Th and 85% for ^226^Ra. Also, the ratio between the two techniques in this study was also much better than the values in Baeza *et al*.^11^, as shown in Table 2. This study furthermore showed the better agreement of measured ^226^Ra(^238^U), ^232^Th, and ^40^K values with the *in-situ* and laboratory HPGe detectors than Dźaluk *et al*.^20^ who used *in-situ* and laboratory HPGe detectors to measure ^226^Ra(^238^U), ^232^Th, and ^40^K in granite in Poland. This is due to the approach employed in this study of validating the homogeneity of the distribution of natural radionuclides in the selected sites using the car-borne system, the flatness of the site, and avoiding measurements on rainy days. From these results, the *in-situ* gamma spectrometer can precisely measure the activity concentrations of ^226^Ra (^238^U), ^232^Th, and ^40^K with good quality assurance.

The natural radionuclides of ^226^Ra (^238^U), ^232^Th, and ^40^K were also measured (in cps unit) with the NaI(Tl) detector in the car-borne system using ROIs of ^40^K, ^238^U (or ^226^Ra (via ^214^Bi)), and ^232^Th (via ^208^Tl) of the γ-ray spectrum calculated using the method described by the IAEA report TECDOC-1363. In order to utilize the car-borne γ-ray spectrum survey over Korea in the radon potential risk mapping program, the cps values of ^226^Ra (^238^U), ^232^Th, and ^40^K should be converted to specific activity concentrations with Bq kg^−1^ unit. This can be done by comparing the relation of simultaneous measurement of these natural radionuclides with the *in-situ* HPGe portable detector and the NaI(Tl) car-borne system.

**Figure 4:**
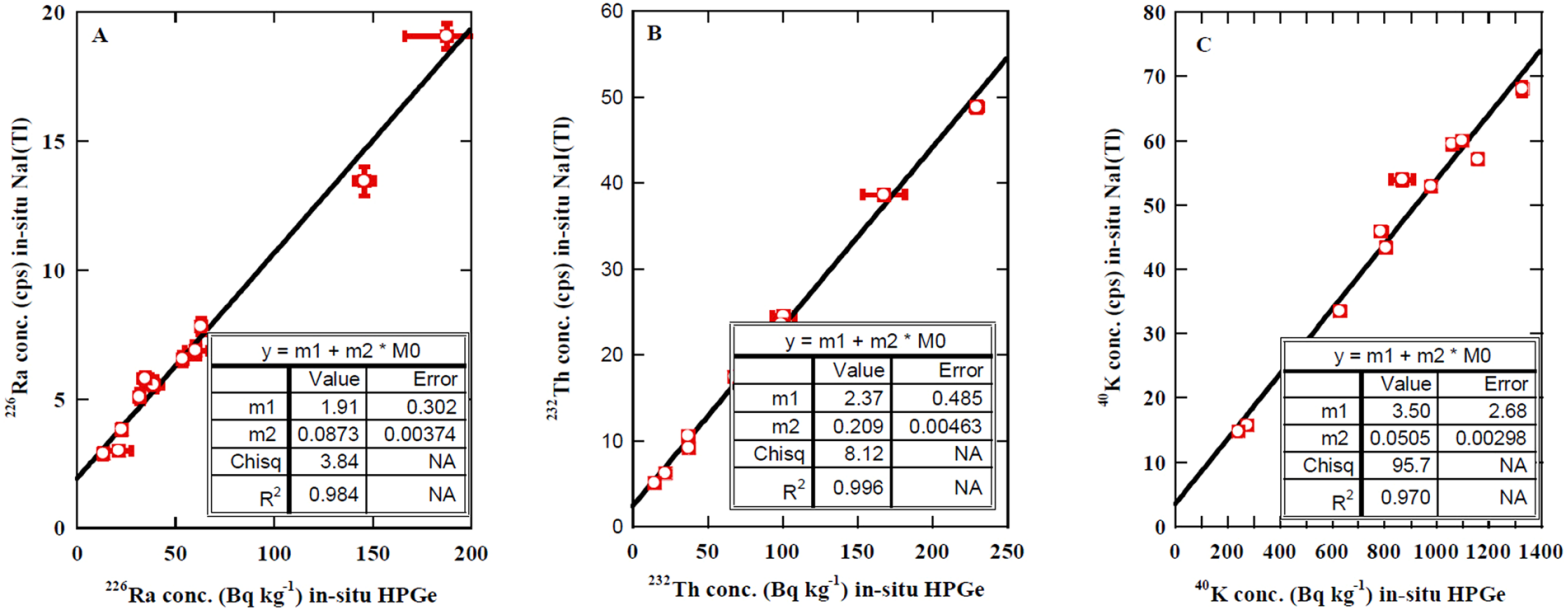
Correlation between the measured activity concentration of ^226^Ra, ^232^Th, and ^40^K using an *in-situ* HPGe detector and NaI(Tl) detector in vehicle survey system.

Figure 4A presents the relation between the measured value of ^226^Ra with the NaI(Tl) detector in the car-borne system and the *in-situ* HPGe detector. A strong correlation clearly appeared with a linear regression coefficient of R^2^ = 0.98 with an equation of y = (0.087 ± 0.004) x + (1.91 ± 0.30); i.e. the measured ^226^Ra in cps with the NaI(Tl) detector equals 0.087 ± 0.004 multiplied by the measured ^226^Ra in Bq kg^−1^ with the *in-situ* HPGe detector plus 1.91 ± 0.30. The concentration of ^226^Ra in Bq kg^−1^ can be estimated from this empirical formula.

Figure 4B shows the correlation between the measured ^232^Th in cps counts with NaI(Tl) in the car-borne system and value obtained with the *in-situ* HPGe detector. One can easily see a strong correlation with a linear regression coefficient of R^2^ = 0.99 with an equation of y = (0.21 ± 0.01) x + 2.37 ± 0.49. This equation will serve as an empirical equation to convert cps counts of ^232^Th value to the specific activity concentration of ^232^Th Bq kg^−1^.

Figure 4C presents the correlation between the measured value of ^40^K in cps counts with the NaI(Tl) and the value obtained with the *in-situ* HPGe detector. A strong correlation appeared. This is confirmed by its linear regression coefficient of R^2^ = 0.97 with an equation of y = (0.051 ± 0.003) x + 3.50 ± 2.68. This equation will serve as an empirical equation to convert cps counts of ^40^K value to the specific activity concentration of ^40^K Bq kg^−1^. It is noteworthy that the gamma spectrum survey of natural radionuclides can be converted to activity concentrations in order to be utilized in the radon potential risk mapping program.

In Fig. 4A–C, the intercepts of 1.91 ± 0.30, 2.37 ± 0.49, and 3.50 ± 2.68 are inferred to be the background of ROI regions of ^226^Ra (1764.5 keV), ^232^Th (2614 keV), and ^40^K (1460.8 keV), respectively, originated from the materials of car, the internal radioactivity of the NaI(Tl) detector, and cosmic rays.

Meanwhile, to convert the cps counts of the NaI(Tl) detector to specific activity concentrations of the radionuclides, IAEA recommends using cylindrical concrete calibration pads containing known concentrations of the radionuclides^9,12^. Four pads (8 m × 0.5 m) are recommended for airborne instruments, while smaller calibration pads (3 m × 0.5 m) are recommended for the calibration of portable gamma-ray spectrometers. Each of the pads is enriched with either K, U or Th. The recommended concentrations are 8% K in the K-pad, 50 ppm U in the U-pad, and 125 ppm Th in the Th-pad. The fourth pad serves as a background pad. In addition, because background radiation is due to the internal radioactivity of the instrument, cosmic radiation, and atmospheric radon, the background is estimated by taking measurements from a small boat (preferably fiberglass) over a river or lake, and at least 200 m from the shore. The shoreline should be flat. Background count rates should be recorded in all energy channels. However, in practice, it is not easy to make the calibration pads and to find a suitable site or boat for determining the background count rate of the system. In addition, the calibration factor estimated on the concrete pads should be corrected by car-body shielding attenuation to apply the car-borne γ-ray spectrometry. Another calibration method is used by means of comparing the potassium, uranium, and thorium window count rates over a calibration site with the ground concentrations of potassium, uranium, and thorium measured with a calibrated portable gamma ray spectrometer^13^. Based on the ground concentrations of the site and the count rates in the three windows, the system sensitivities were determined. To carry out the calibration using this method, one should previously measure these nuclides, check the homogeneity of the concentration distributions in the calibration site, and determine the background count rate of the car-borne γ-ray spectrometry system. Therefore, the method in this study is expected to be used to calibrate a car-borne measurement system equipped with a NaI(Tl) detector without previous characterization of the calibration site and background measurement of the detector.

### Evaluation of gamma radiation dose rate

#### Calculation of absorbed dose rate in air from measured values of natural radionuclides

**Figure 5:**
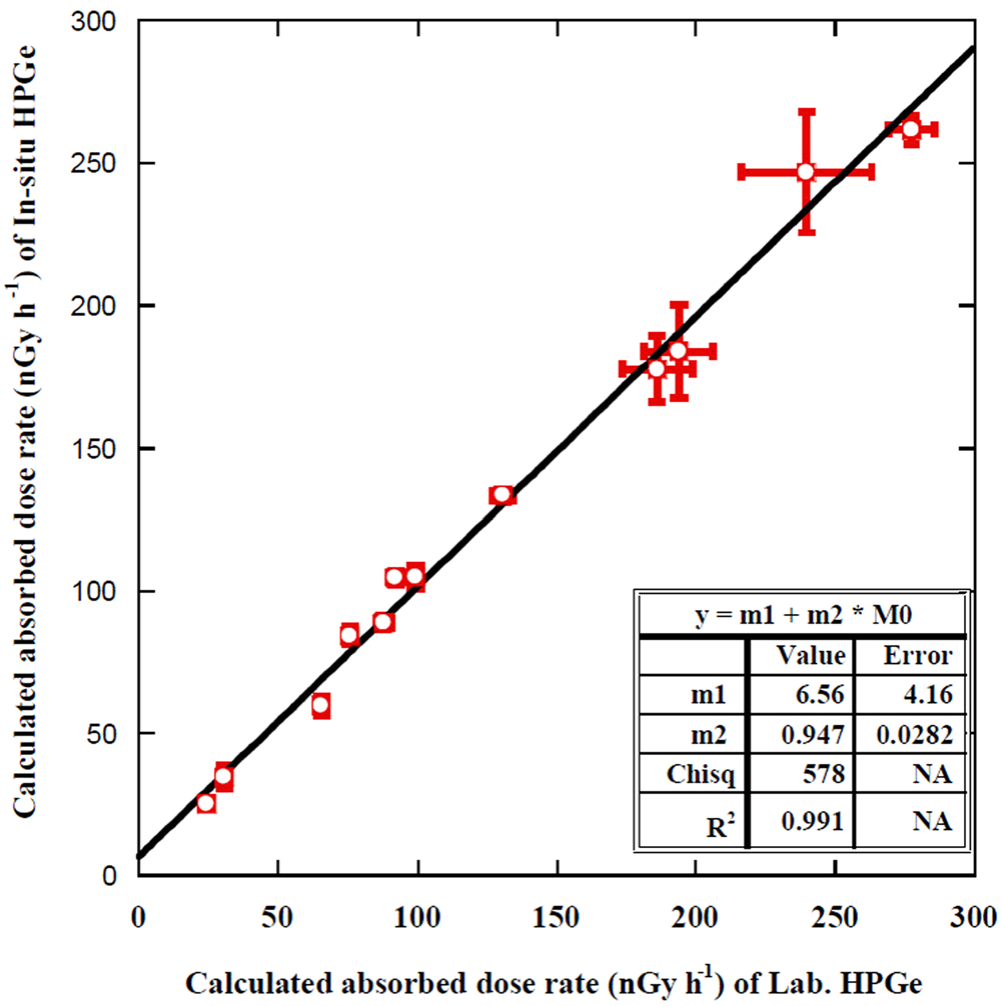
The correlation between calculated absorbed dose rates with *in-situ* and laboratory HPGe detector.

The absorbed gamma radiation dose rate in air at 1 m height above the ground can be calculated from the measured values of natural radionuclides of ^226^Ra (^238^U), ^232^Th, and ^40^K in soil (*in-situ* and laboratory HPGe detectors). A homogenous distribution of natural radionuclides in the soil and flatness of the soil surface was assumed. The absorbed dose rate (nGy/h) in air can be calculated from the well known Beck formula (Eq. 2)^4,5,8,11,21^ using dose conversion factors reported by UNSCEAR^3^

2 $$D(nGy/h)=(0.462{A}_{Ra})+(0.604{A}_{Th})+(0.0417{A}_{K}),$$

where *A*_Ra_, *A*_Th_, and *A*_K_ are the activity concentrations of ^226^Ra, ^232^Th, and ^40^K, respectively. As shown in Table 3, the absorbed dose rate varied from 25.0 ± 1.0 to 261 ± 5 nGy h^−1^ with an average value of 125 ± 6 nGy h^−1^. The absorbed dose rate in Jeju Island was lower among all the selected sites while Chuncheon was higher, because of their geological origin of Quarternary Basalt/trachyte and Jurassic granite, respectively. The average value agreed with the literature data of radiation dose in South Korea^19^. Figure 5 presents the relation of the calculated dose rate from the measured radionuclides values with the *in-situ* and the laboratory gamma spectrometers. A strong correlation clearly appeared with a linear regression coefficient of R^2^ = 0.99 with an equation of y = (0.947 ± 0.028) x + (6.56 ± 4.16), which is much better than published data for the same relation. For instance, Baeza *et al*.^11^ reported that the correlation of absorbed radiation dose rate obtained from *in-situ* and laboratory gamma spectrometers (the same techniques as used in this study) was 0.95. This is due to the validation of the homogeneous distribution of natural radionuclides in the studied site and its flatness.

**Table 3:** Calculated and measured absorbed γ-rays dose rate.

Site code	Calculated absorbed γ-rays dose rate (nGy h^−1^)			Measured absorbed γ-rays dose rate (nSv h^−1^)
	*In-situ*	Laboratory	*In-situ*/Lab.	HPIC
JI-1	59.6 ± 3.2	65.6 ± 1.9	0.91 ± 0.05	93.8 ± 3.3
JI-2	34.6 ± 3.9	30.9 ± 1.5	1.12 ± 0.14	63.1 ± 2.2
JI-3	25.0 ± 1.0	24.5 ± 1.0	1.02 ± 0.06	56.3 ± 2
CI-1	105 ± 2	92.3 ± 2.5	1.13 ± 0.04	139 ± 5
CI-2	184 ± 16	194 ± 12	0.95 ± 0.10	215 ± 8
CI-3	133 ± 2	131 ± 4	1.02 ± 0.03	166 ± 6
GI-1	88.7 ± 2.4	88.0 ± 2.7	1.01 ± 0.04	134 ± 5
CC-1	261 ± 5	278 ± 8	0.94 ± 0.03	272 ± 10
CC-2	105 ± 4	99.6 ± 2	1.05 ± 0.05	146 ± 5
CC-3	84.3 ± 3	76.1 ± 1.3	1.11 ± 0.04	134 ± 5
WB-1	177 ± 12	186 ± 13	0.95 ± 0.09	199 ± 7

All uncertainties were combined uncertainties at k = 1 given by the gamma spectroscopy programs of Gene2000 and Gamma Vision software.

#### Measurement of gamma radiation dose rate with HPIC

**Figure 6:**
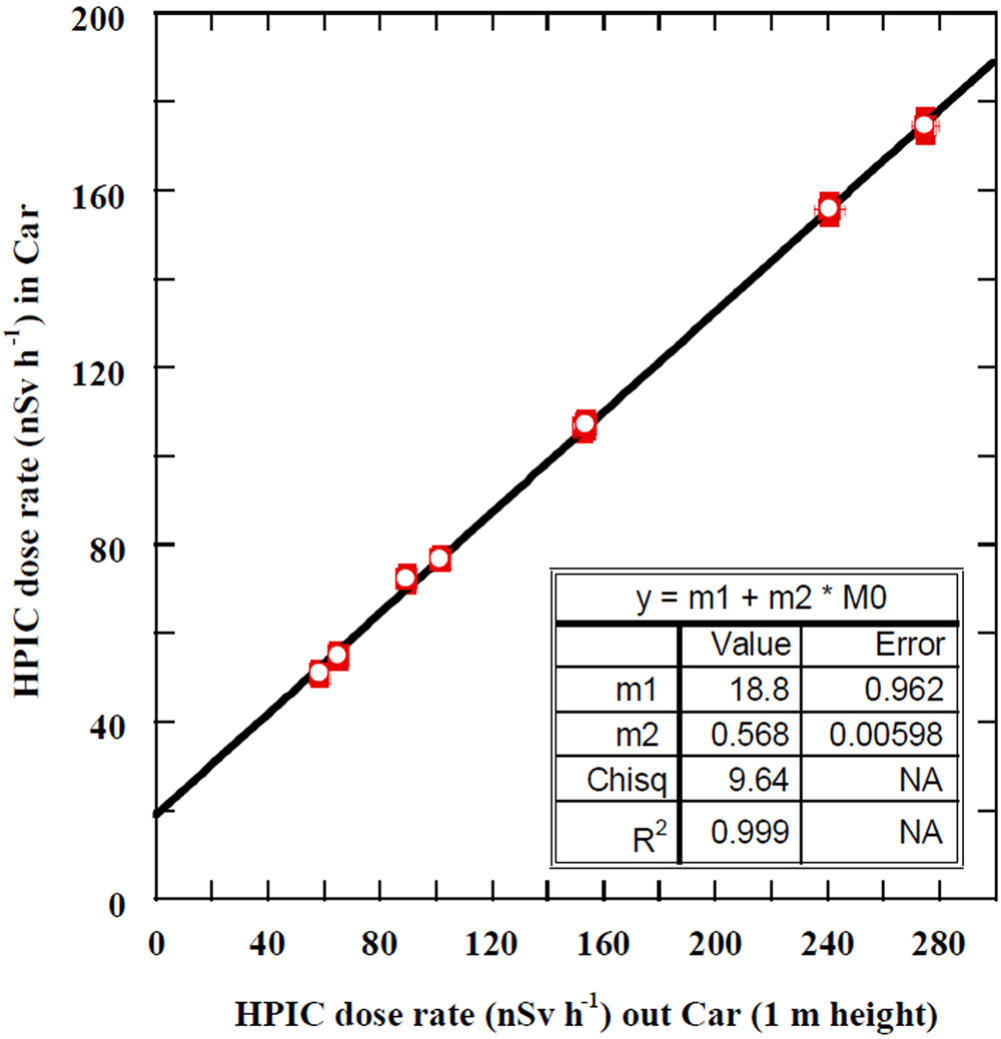
Correlation between absorbed dose rates inside and outside the car in order to estimate the car’s materials shielding factor.

Because this work is highly concerned with the precision and quality assurance of obtained data, the gamma radiation dose rate due to the presence of natural radionuclides in soil was also measured with an HPIC, which has high sensitivity, stability, and reliability to γ-rays measurement. The HPIC can measure the gamma radiation dose rate up to 1 Sv/h with a calibration accuracy of ±3.5% (manufacturer manual). Before using the HPIC in the car-borne system in measuring the dose rate of emitted γ-rays from natural radionuclides over the selected sites, the attenuation shield effect factor of the car body material was evaluated, as shown in Fig. 6. For measuring the shield effect factor, radiation dose rates of eight sites were measured while the HPIC was inside and outside the car at a height of 1 m above the flat ground. Figure 6 presents the relation of the measured radiation dose rate with the HPIC inside and outside the car. A strong correlation was observed with a linear regression coefficient of R^2^ = 0.999 with an equation of y = (0.57 ± 0.01) x + (18.8 ± 0.96). The y-intercept, 18.8 ± 0.96, reflects the background radiation due to the materials of the car body, other measurement systems, operating computers, etc. The values of the radiation dose rate of 11 sites measured while the HPIC was inside the car were then corrected using the above equation with a shielding factor of 0.57 ± 0.01.

**Figure 7:**
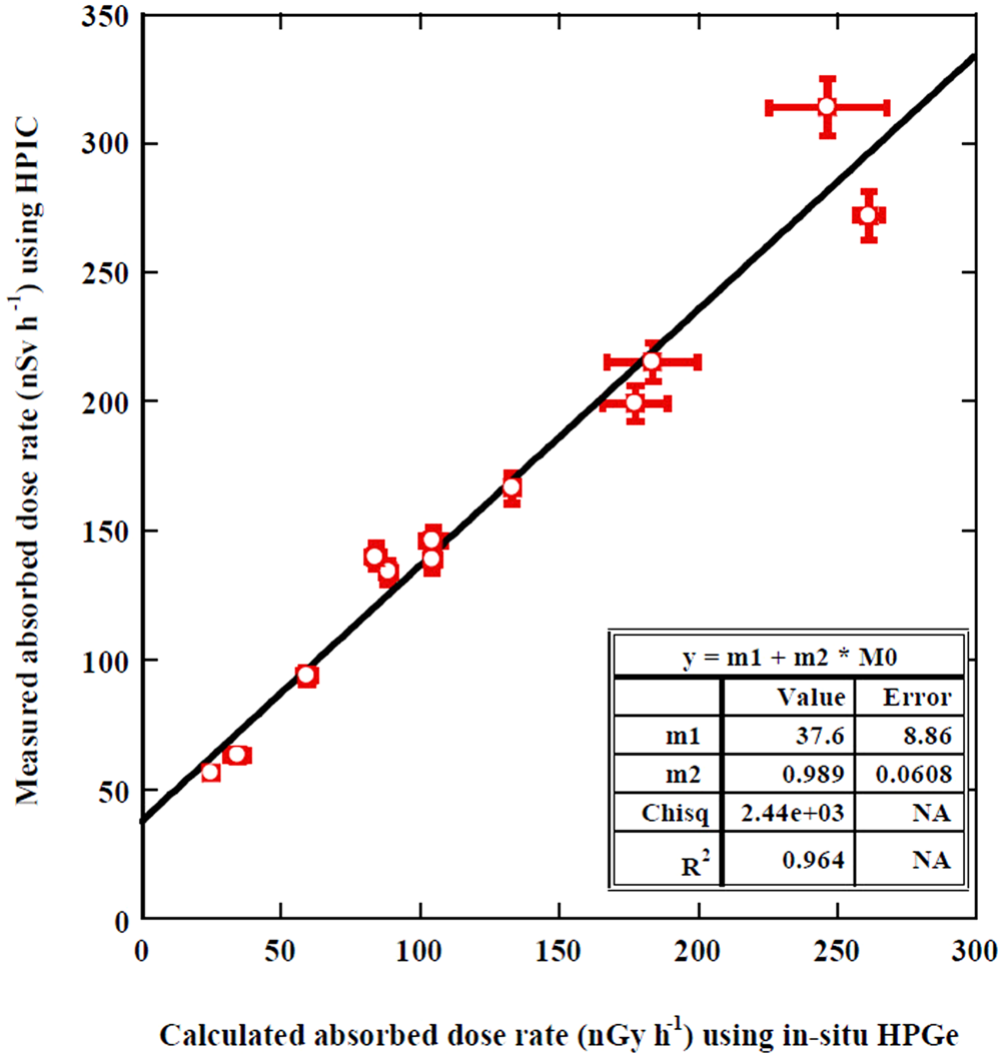
Correlation between calculated absorbed radiation dose rates from radionuclides measured values with the *in-situ* HPGe detector and measured absorbed radiation dose rate using HPIC.

The radiation dose rate (measured with the HPIC) varied from 56.3 ± 2 to 215 ± 8 with a mean value of 142.52 ± 5.97 nSv h^−1^, respectively, as shown in Table 3. It can be observed that the gamma radiation absorbed dose rate in Jeju Island was lower than at all the other sites while it was higher in the Chuncheon site due to its geological origin, as confirmed from all the measurement values. The gamma radiation dose rate measured with the HPIC was compared with the air absorbed dose rate calculated from the measured radionuclides with the *in-situ* portable HPGe detector (the two measurement systems of the HPIC and the *in-situ* HPGe detector were used in the same conditions for every parameter of site flatness, radionuclides distribution, soil moisture, and air temperature). Figure 7 shows the strong correlation between the measured and calculated radiation dose rates with a linear regression coefficient of R^2^ = 0.96 with an equation of y = (0.99 ± 0.06) x +(37.6 ± 8.86) intercepting the y-axis at 37.6 nSv h^−1^ which reflects the approximate value of the radiation dose rate of cosmic rays due to the ability of the HPIC to detect these rays^22^. This value is almost similar to the average value (33.4 nSv/h) of cosmic rays in Korea at the ground level^23^ (considering the experimental error). The x-axis presents the radiation dose rate of natural radionuclides only which were measured by a HPGe detector while the y-axis presents the radiation dose rate of natural radionuclides and cosmic rays. It was reported that the worldwide radiation dose of cosmic rays at sea level is 31 nSv/h and that this value increases with altitude^3^. This study confirms a strong correlation between the measured and calculated doses as seen from the correlation coefficient of R^2^ = 0.96 and equal of both units of Gy h^−1^ and Sv h^−1^ as in an equation of y = (0.99 ± 0.06) x + (37.6 ± 8.86) as reported in the literature^24^. Other studies reported that the correlation between measured and calculated radiation dose varied from weak to strong. For instance, Losana *et al*.^25^ reported the correlation between the measured radiation dose with HPIC and the calculated value of measured natural radionuclides with the *in-situ* HPGe detector (as in the present study) was within 20%. Karunakara *et al*.^4^ reported that the measured dose with a survey meter and TLD with a calculated dose from radionuclide values were R = 83% and R = 52%, respectively. Huang *et al*.^6^ reported that the dose measured with a portable survey meter and TLD with a calculated dose of the radionuclides value were R = 97%. From all of the published data, we can confirm precise measurement of the natural radionuclides and radiation dose.

## Conclusion

The specific activity concentrations of natural radionuclides in soil samples from 11 sites over Korea were precisely measured with several γ-ray spectroscopy techniques of *in-situ* HPGe and Na(Tl) and conventional laboratory HPGe detectors. A strong correlation between the measured radionuclides with *in-situ* and laboratory gamma spectrometers was observed, which indicates that *in-situ* HPGe detector can precisely measure the activity concentrations of ^226^Ra (^238^U), ^232^Th, and ^40^K at the ground level of the soil. Also, a strong correlation between *in-situ* HPGe and Na(Tl) detectors was observed and employed to convert the cps counts of Na(Tl) of ^226^Ra (^238^U), ^232^Th, and ^40^K to specific activity concentrations with Bq kg^−1^in order to utilize the gamma count survey into a radon potential risk mapping program. Moreover, the radiation dose rate due to the presences of natural radioactive nuclides of ^226^Ra (^238^U), ^232^Th, and ^40^K in soil was measured with HPIC after the shielding factor was estimated. On the other hand, radiation dose rate was calculated from the measured values of natural radionuclides. Both methods show a strong correlation with a correlation factor of R^2^ = 0.93 with an equation of y = 0.982 x + 36.3 intercepting the y-axis at nSvh^−1^ which approximately reflects a radiation dose of cosmic rays of 36.3 nSv h^−1^. This study confirms, from a comparison with several techniques and published data, that natural radionuclides and their corresponding radiation dose with our systems were precisely evaluated.
